# Non-steroidal FXR agonist cilofexor improves cholestatic liver injury in the *Mdr2*^-/-^ mouse model of sclerosing cholangitis

**DOI:** 10.1016/j.jhepr.2023.100874

**Published:** 2023-08-03

**Authors:** Claudia D. Fuchs, Natalie Sroda, Hubert Scharnagl, Ruchi Gupta, Wesley Minto, Tatjana Stojakovic, John T. Liles, Grant Budas, David Hollenback, Michael Trauner

**Affiliations:** 1 Hans Popper Laboratory of Molecular Hepatology, Division of Gastroenterology and Hepatology, Department of Internal Medicine III, Medical University of Vienna, Austria; 2 Gilead Sciences, Inc., Foster City, CA, USA; 3 Clinical Institute of Medical and Chemical Laboratory Diagnostics, Medical University of Graz, Austria; 4 Clinical Institute of Medical and Chemical Laboratory Diagnostics, University Hospital Graz, Austria

**Keywords:** Bile acid signaling, Inflammation Fibrosis, FXR, FGF15/19 Bile acid metabolism

## Abstract

**Background & Aims:**

The nuclear receptor farnesoid X receptor (FXR) is a key regulator of hepatic bile acid (BA) and lipid metabolism, inflammation and fibrosis. Here, we aimed to explore the potential of cilofexor (GS-9674), a non-steroidal FXR agonist, as a therapeutic approach for counteracting features of cholestatic liver injury by evaluating its efficacy and mechanisms in the *Mdr2/Abcb4* knockout (^-/-^) mouse model of sclerosing cholangitis.

**Methods:**

FVB/N wild-type and *Mdr2*^*-/-*^ or BALB/c wild-type and *Mdr2*^*-/-*^ mice were treated with 0, 10, 30 or 90 mg/kg cilofexor by gavage every 24 h for 10 weeks. Serum biochemistry, gene expression profile, hydroxyproline content, and picrosirius red and F4/80 immunostaining, were investigated. Bile flow, biliary bicarbonate and BA output, and hepatic BA profile, were assessed.

**Results:**

Cilofexor treatment improved serum levels of aspartate aminotransferase, alkaline phosphatase as well as BAs in *Mdr2*^*-/-*^ animals. Hepatic fibrosis was improved, as reflected by the reduced picrosirius red-positive area and hydroxyproline content in liver sections of cilofexor-treated *Mdr2*^*-/-*^ mice. Intrahepatic BA concentrations were lowered in cilofexor-treated *Mdr2*^*-/-*^ mice, while hepatobiliary bile flow and bicarbonate output were increased.

**Conclusion:**

Collectively the current data show that cilofexor treatment improves cholestatic liver injury and decreases hepatic fibrosis in the *Mdr2*^*-/-*^ mouse model of sclerosing cholangitis.

**Impact and implications:**

Treatment with cilofexor, a non-steroidal farnesoid X receptor (FXR) agonist, improved histological features of sclerosing cholangitis, cholestasis and hepatic fibrosis in the *Mdr2*^*-/-*^ mouse model. These findings indicate, that pharmacological stimulation of intestinal FXR-mediated gut-liver signaling, via fibroblast growth factor 15 (thereby reducing bile acid synthesis), may be sufficient to attenuate cholestatic liver injury in the *Mdr2*^*-/-*^ mouse model of sclerosing cholangitis, thus arguing for potential therapeutic properties of cilofexor in cholestatic liver diseases.

## Highlights

• Treatment with cilofexor improves cholestatic liver injury and decreases hepatic fibrosis in the *Mdr2*^*-/-*^ mouse model.
• Cilofexor significantly reduced the expression of markers of activated hepatic stellate cells, such as *αSma*, *Desmin* and *Pdgfrβ.*
• Cilofexor treatment reduced expression of *Cyp7a1*, resulting in a significant decrease in serum and intrahepatic bile acid levels.

## Graphical abstract

**Figure:**
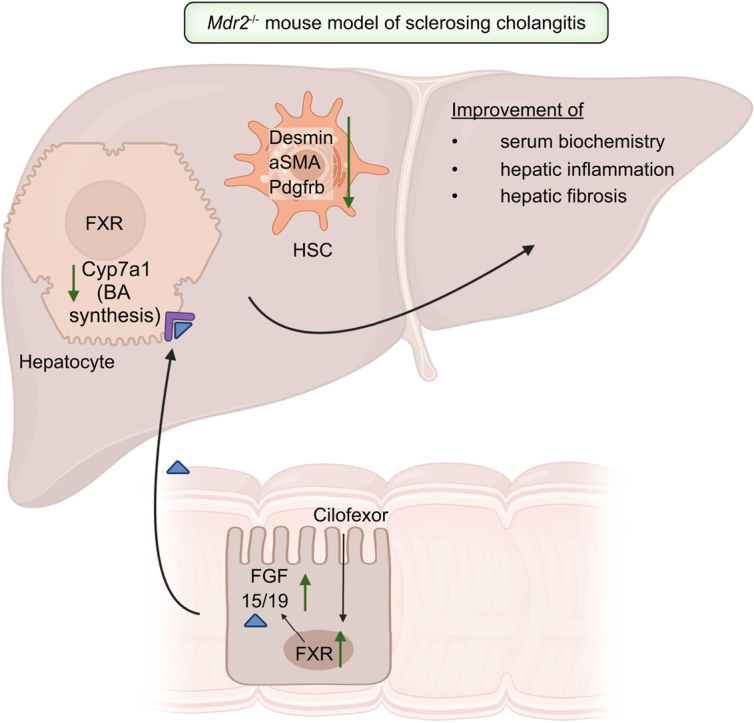

## Introduction

Chronic cholangiopathies such as primary sclerosing cholangitis (PSC) are diseases with huge unmet medical need. Fibrosis is a key feature of PSC that can progress to cirrhosis and ultimately end-stage liver disease.^1^ Available pharmacological strategies have limited efficacy and novel therapies are eagerly awaited.^2^ The bile acid-activated farnesoid X receptor (FXR, NR1H4) has emerged as a promising therapeutic target[3], [4], [5] as it orchestrates key processes that may counteract or at least ameliorate cholestasis. FXR agonists broadly control hepatic bile acid (BA) and cholesterol metabolism, thereby limiting hepatocellular retention of potentially toxic BAs, protecting the bile duct epithelium from intrinsically toxic bile, and suppressing inflammation, which may ultimately lead to reduced fibrosis. Several *in vivo* studies using the steroid-based FXR agonist, obeticholic acid (OCA, aka 6-ethyl-CDCA or INT-747), have shown hepatoprotective effects in animal models of cholestasis and hepatic fibrosis.^6^^,^^7^ Based on these findings clinical trials of OCA in patients with primary biliary cholangitis (PBC) or PSC were conducted,[8], [9], [10], [11] with OCA gaining approval for PBC. The success of OCA has spurred development of non-steroidal FXR agonists, as each new structure may produce a unique FXR transcriptional repertoire and differing plasma/tissue ratios.^12^ This in turn may help manage some of the safety concerns (*e.g.* pruritus and cholesterol changes) endemic to the class.^9^^,^^13^^,^^14^ Indeed, data from a recent placebo-controlled study with the non-steroidal agonist, cilofexor, showed that administration at 30 mg or 100 mg q.d. was well tolerated in patients with PSC and led to significant improvements in liver biochemistry and markers of cholestasis.^15^ In the current study, we aimed to gain insight into mechanistic aspects of cilofexor and to evaluate the potential anti-cholestatic, anti-inflammatory and anti-fibrotic effects driven by FXR agonism in the *Mdr2*^*-/-*^ mouse, an established model of sclerosing cholangitis.

## Materials and methods

### Animals experiments

Male FVB/N (wild-type or *Mdr2*^*-/-*^) or male and female BALB/cJ (wild-type and *Mdr2*^*-/-*^) mice were housed in a 12 h light dark cycle. Animals had unrestricted access to water and food. Cilofexor at 0 (vehicle: 0.5% carboxymethylcelluose and 1% ethanol in Tris Buffer, pH: 8), 10, 30, or 90 mg/kg was administered via gavage every 24 h over a time-period of 10 weeks starting from week 6 (Fig. S1). For tissue collection, mice were euthanized at 2 h post dose, when we typically observe maximal gene expression changes in the ileum and liver (data not shown) and is consistent with a half-life of 4 h. For the BALB/cJ *Mdr2*^*-/-*^ mice, a natural history study showed no statistical difference in alkaline phosphatase (ALP), hydroxyproline or picrosirius red (PSR) between sexes at 6, 8, 12 or 16 weeks (data not shown), although previously^16^ a sex difference in liver hydroxyproline content in BALB/c cAnNCrl *Mdr2*^*-/-*^ mice has been demonstrated. There were also no sex differences in plasma or liver exposure to cilofexor. Therefore, to use the minimal number of animals possible both sexes from the Gilead breeding colony were placed on study and their data combined. This animal study was approved by the Animal Ethics Committee of the Medical University of Vienna and the Federal Ministry of Science, Research and Economy and was performed according to the Animal Research: Reporting of *In Vivo* Experiments (ARRIVE) guidelines or the U.S. Department of Agriculture’s Animal Welfare Act (9 CFR Parts 1, 2, and 3), the Guide for the Care and Use of Laboratory Animals (Institute for Laboratory Animal Research, The National Academies Press, Washington, D.C.), and the National Institutes of Health, Office of Laboratory Animal Welfare.

### Bile flow measurement

Bile flow was measured as described previously.^17^ After 10 weeks of substance administration the common bile duct was ligated and the gallbladder was cannulated. After a 5 min equilibration period, bile was collected in pre-weighted tubes for 30 min. Bile flow was determined gravimetrically and normalized to liver weight. Biliary bicarbonate concentrations were measured with a blood gas analyzer.

### Liver bile acid measurement

Liver BAs were analyzed using an Agilent 1290 Infinity/Sciex QTRAP 6500 LC-MS/MS system equipped with a C18 reverse phase UHPLC column (Metabolon, North Carolina, USA).

### Liver histology and biochemical analysis

For conventional light microscopy, livers were fixed in 4% neutral buffered formaldehyde solution for 24 h, embedded in paraffin, and stained with H&E or PSR^18^ and F4/80^19^ as described. Hydroxyproline was measured biochemically and normalized to liver weight using cryo-powdered livers as described.^20^

### Serum analysis

Blood was collected during tissue harvesting and centrifuged for 15 min at 4,500 rpm. Serum was stored at -80 °C until analysis. Levels of transaminases (aspartate aminotransferase [AST]; alanine aminotransferase [ALT]), ALP, total cholesterol, triglycerides (Roche Diagnostics, Mannheim, Germany), fatty acids (Wako Chemicals GmbH, Neuss, Germany) and BAs (DiaSys Diagnostic Systems GmbH, Holzheim, Germany) were measured using enzymatic methods according to the manufacturer's instructions or on an Olympus AU400e Clinical Chemistry Analyzer (Beckman Coulter, Inc.).

### RNA isolation and qRT-PCR analysis

Tissues were snap frozen in prechilled 2-methylbutane and stored in liquid nitrogen. RNA was extracted from a standardized liver piece using TRIzol reagent (Invitrogen, Carlsbad, CA) according to manufacturer’s instructions. 1.5 μg of RNA was used for complementary DNA synthesis using random hexamer primer (Applied Biosystems) and Superscript II reverse transcriptase (Invitrogen, Carlsbad, CA) according to manufacturer’s instructions. 1:20 dilution of the cDNA was used for qRT-PCR (quantitative reverse-transcription PCR) using SYBR Green Master Mix (Applied Biosystems) or Fast Advance Master Mix (TaqMan) and was performed using the AB7900 or QuantStudio 6 Flex Real-Time PCR system (Applied Biosystems). Reactions were performed in duplicates and relative mRNA levels were quantified using a calibration dilution curve normalized to the housekeeping genes. mRNA levels were normalized to *36b4, Gapdh,* or the geomean of *B2m, Gapdh, Hprt1, Pgk1,* and *Rpl13a* as housekeeping gene which did not vary between groups.

### Liver cytokine measurement

Liver cytokines were analyzed using the MILLIPLEX® Mouse Cytokine/Chemokine Magnetic Bead Panel (DC3 Therapeutics, South San Francisco, USA).

### Statistical analysis

Results were evaluated using SPSS V.27.0 or GraphPad Prism 9.3.0. Statistical analysis was performed using Student’s unpaired two-tailed *t* test or one-way ANOVA. Data are reported as means of 5-15 animals per group ±SD. A *p* value ≤0.05 was considered statistically significant.

## Results

### Cilofexor treatment improves hepatic fibrosis in the FVB/N *Mdr2*^*-/-*^ mouse model of sclerosing cholangitis

**Fig. 1:**
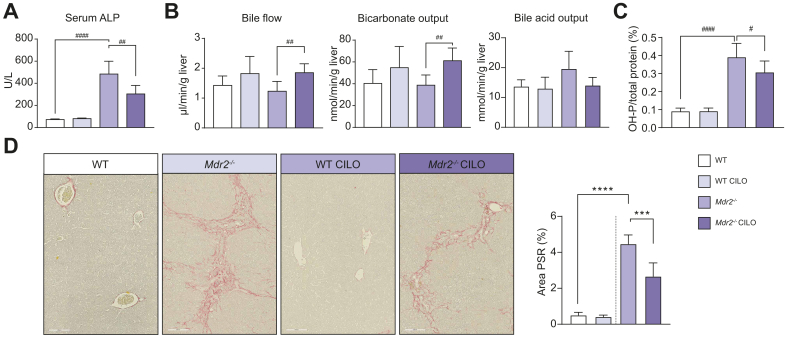
FVB/N *Mdr2^-/-^* display reduced fibrosis after 10 weeks of treatment with cilofexor. FVB/N WT or *Mdr2^-/-^* (n = 5-7) at 6 weeks of age we treated with vehicle or cilofexor (90 mg/kg) for 10 weeks. (A) Serum ALP was increased in the *Mdr2^-/-^* mice but attenuated by treatment with cilofexor. (B) Hepatic bile flow and bicarbonate output was unchanged between WT and *Mdr2^-/-^* mice but increased with cilofexor treatment. BA output was tendentially reduced in *Mdr2^-/-^* mice treated with cilofexor compared to *Mdr2^-/-^* vehicle treated mice. (C) Hepatic hydroxyproline content as well as (D) percent PSR staining were reduced with cilofexor treatment compared to *Mdr2^-/-^* vehicle treated mice. Results are expressed as mean ± SD. ^#^*p* < 0.05, ^##^*p* < 0.01, ^###^*p* < 0.001, ^####^*p* < 0.0001 by unpaired t-test. Abbreviations: WT, wild type; *Mdr2^-/-^,* multi-drug resistance protein 2 knock out; ALP, alkaline phosphatase; PSR, picrosirius red.

ALP, a biochemical marker of cholestasis, was reduced in *Mdr2*^*-/-*^ mice by cilofexor therapy (Fig. 1A), while serum levels of liver transaminases ALT and AST, as well as BAs, remained unchanged (Fig. S2). Bile flow as well as bicarbonate output were increased in the cilofexor-treated *Mdr2*^*-/-*^ mice compared to controls (Fig. 1B). Hepatobiliary BA output was tendentially reduced in cilofexor-treated *Mdr2*^*-/-*^ mice compared to controls (Fig. 1B). While gene expression of inflammatory markers *Ccl5* and *Tnfα* remained unchanged among the groups (Fig. S3), hepatic hydroxyproline content as well as PSR (as a marker of hepatic fibrosis)-positive areas were reduced in liver sections of cilofexor-treated *Mdr2*^*-/-*^ mice in comparison to *Mdr2*^*-/-*^ control animals (Fig. 1C and D). Together these data indicate that cilofexor treatment improves hepatic fibrosis (but not inflammation) in FVB/N *Mdr2*^*-/-*^ mice.

### Cilofexor treatment improves hepatic fibrosis and inflammatory markers in the BALB/cJ *Mdr2*^-/-^ mouse model of sclerosing cholangitis

**Fig. 2:**
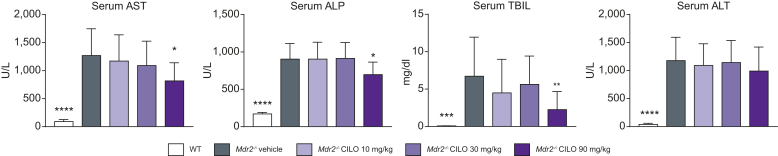
BALB/cJ *Mdr2^-/-^* have increased serum markers of liver injury that improved with cilofexor treatment. BALB/cJ *Mdr2^-/-^* (n = 15) at 6 weeks of age were treated with vehicle or cilofexor (10, 30, or 90 mg/kg) for 10 weeks. Serum AST, ALP, and total bilirubin, but not ALT, were decreased at 90 mg/kg cilofexor. Results are expressed as mean ± SD. ∗ *p* < 0.05, ∗∗*p* < 0.01, ∗∗∗*p* < 0.001, ∗∗∗∗ *p* < 0.0001 *vs*. *Mdr2^-/-^* vehicle by one-way ANOVA. Abbreviations: WT, wild type; *Mdr2^-/-^*, multi-drug resistance protein 2 knock out; ALT, Alanine amino transferase; AST, Aspartate amino transferase; ALP, alkaline phosphatase, TBIL, total bilirubin.

**Fig. 3:**
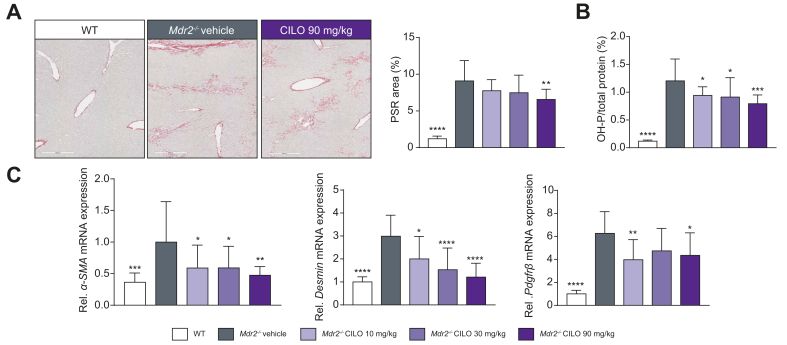
Cilofexor treatment decreases markers of hepatic fibrosis and activated hepatic stellate cells in the BALB/cJ *Mdr2^-/-^* mice. (A) Representative PSR images and quantitative analysis show reduction in percent PSR staining with cilofexor treatment in a dose dependent manner in line with (B) reduced hepatic hydroxyproline content and (C) hepatic gene expression of markers for activated hepatic stellate cells *aSma, Desmin and Pdgfrb*. Results are expressed as mean ± SD as per Fig. 2. ^∗^*p* < 0.05, ^∗∗^*p* < 0.01, ∗∗∗*p* < 0.001, ∗∗∗∗*p* < 0.0001 *vs. Mdr2^^-/-^^* vehicle by one-way ANOVA. Abbreviations: WT, wild type; *Mdr2^-/-^,* multi-drug resistance protein 2 knock out; CILO, cilofexor; PSR, picrosirius red; *α-Sma*, alpha smooth muscle actin.

**Fig. 4:**
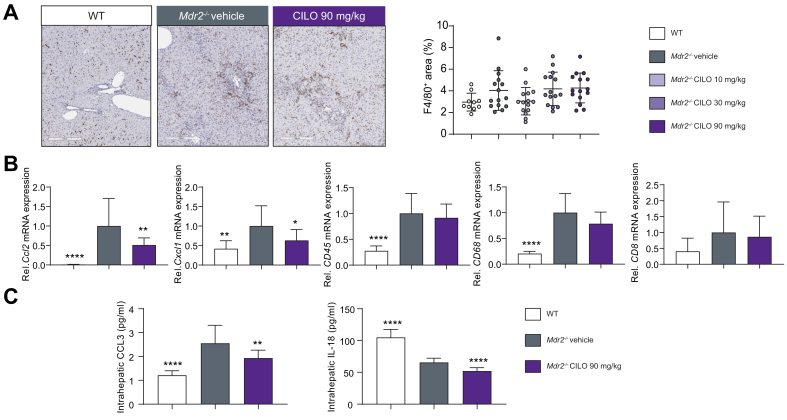
Cilofexor treatment reduces inflammatory markers in the liver of BALB/cJ *Mdr2^-/-^ mice.* (A) Representative F4/80 images and quantitative analysis revealed no difference among the groups. (B) Liver mRNA levels of inflammatory markers *Ccl2* and *Cxcl2* as well as (C) liver cytokines IL-18 and CCL3 were reduced after treatment of cilofexor at 90mg/kg in the Mdr2^-/-^ mice. Cilofexor treatment did not affect the mRNA levels of *Cd45*, *Cd68*, or *Cd8*. Results are expressed as mean ± SD as per Figure 2. ^∗^*p* < 0.05, ^∗∗^*p* < 0.01, ∗∗∗*p* < 0.001, ∗∗∗∗*p* < 0.0001 *vs. Mdr2^^-/-^^* vehicle by one-way ANOVA. Abbreviations: WT, wild type; *Mdr2^-/-^,* multi-drug resistance protein 2 knock out; CILO, cilofexor; *Ccl2*, chemokine (C-C motif) ligand 2; *Cxcl1*, chemokine (C-X-C motif) ligand 1; CCL3, Chemokine (C-C motif) ligand 3; IL-18, Interleukin-18.

Since BALB/cJ *Mdr2*^*-/-*^ mice develop much more severe liver injury than FVB/N *Mdr2*^-/-^ mice (indicated by markers of fibrosis, liver enzymes and serum BAs, Table S1),^16^ we assessed whether cilofexor is also beneficial in this mouse model. mRNA expression levels of intestinal *Fgf15, Shp, Ostα* and *Ostβ* were significantly increased in a dose-dependent manner in BALB/cJ *Mdr2*^*-/-*^ mice treated with 10, 30, or 90 mg/kg cilofexor daily (Fig. S4). Interestingly, mRNA expression of hepatic *Shp* and *Fgf15* was only increased in animals challenged with 90 mg/kg cilofexor. Accordingly, mRNA levels of *Cyp7a1* were only reduced with 90 mg/kg cilofexor (Fig. S4). At a dose of 90 mg/kg, cilofexor significantly decreased serum levels of AST, ALP and total bilirubin (TBIL) compared to vehicle in *Mdr2*^*-/-*^ mice, although ALT was not changed (Fig. 2). Of note, neither 10 mg/kg nor 30 mg/kg cilofexor had an effect on serum levels of the aforementioned markers (Fig. 2). PSR-positive areas were reduced in liver sections of BALB/cJ *Mdr2*^*-/-*^ mice treated with 90 mg/kg cilofexor (Fig. 3A). Of particular interest, all three cilofexor dose levels significantly lowered liver hydroxyproline content (Fig. 3B). Accordingly, mRNA expression of markers for activated hepatic stellate cells (HSCs), such as *αSma*, *Desmin* and *Pdgfrβ,* were significantly lowered due to cilofexor treatment (Fig. 3C). Regarding hepatic inflammation, the amount of F4/80+ cells was investigated (Fig. 4A). While none of the cilofexor doses tested led to reduced F4/80+ cell numbers, 90 mg/kg cilofexor decreased the mRNA levels of *Ccl2* and *Cxcl1* in the liver but did not affect the mRNA levels of *Cd45, Cd68,* or *Cd8* (Fig. 4B). Treatment with cilofexor at a dose of 90 mg/kg lowered the level of the liver cytokine CCL3 (Fig. 4C). Of note, the proinflammatory liver cytokine IL-18 (downregulated in the *Mdr2*^*-/-*^ animals at baseline) is decreased even further due to cilofexor treatment (Fig. 4C).

### Cilofexor treatment reduces serum and intrahepatic BA levels in the BALB/cJ *Mdr2*^*-/-*^ mouse model of sclerosing cholangitis

**Fig. 5:**
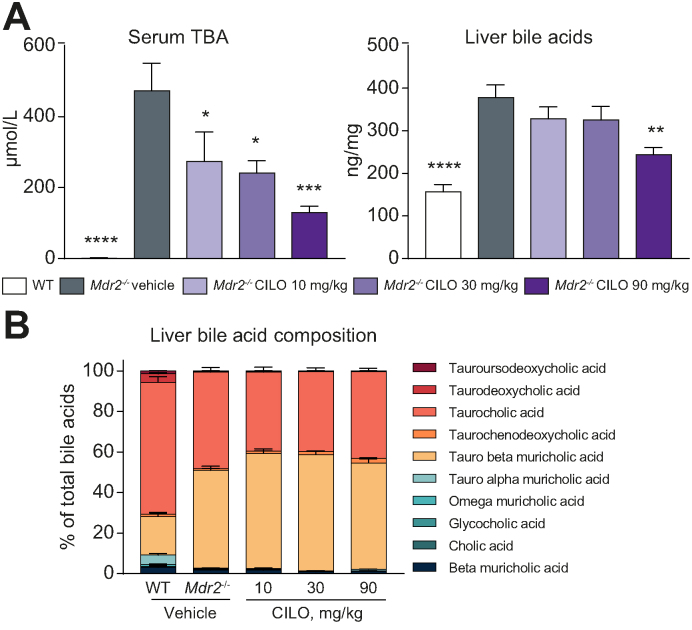
Cilofexor prevents bile acid accumulation in the serum and liver of BALB/cJ *Mdr2^-/-^* mice. (A) Serum BAs were reduced after treatment with cilofexor at all dose levels. Liver BA were reduced at 90 mg/kg cilofexor but (B) bile acid composition remained unchanged. Results are expressed as mean ± SD as per Figure 2. ^∗^*p* < 0.05, ^∗∗^*p* < 0.01, ∗∗∗*p* < 0.001, ∗∗∗∗*p* < 0.0001 *vs. Mdr2^^-/-^^* vehicle by one-way ANOVA. Abbreviations: WT, wild type; *Mdr2^-/-^,* multi-drug resistance protein 2 knock out; CILO, cilofexor; TBA, total BAs.

The reduction of liver *Cyp7a1* mRNA expression seen in BALB/cJ *Mdr2*^*-/-*^ mice treated with 90 mg/kg cilofexor (Fig. S4B) is also reflected by reduced serum and intrahepatic BA levels in these mice compared to vehicle-treated *Mdr2*^*-/-*^ control mice (Fig. 5A and B). Of particular interest, despite unchanged mRNA levels of *Cyp7a1* in *Mdr2*^*-/-*^ mice treated with 10 or 30 mg/kg cilofexor, serum BA levels are also reduced in these groups (Fig. 5A). Despite a significant reduction of intrahepatic BA concentration in *Mdr2*^*-/-*^ mice challenged with 90 mg/kg cilofexor (Fig. 5B), the relative BA composition remained unchanged among all groups even though the absolute levels of taurocholic acid and tauro β-muricholic acid were reduced (Fig. S5). Notably tauro β-muricholic acid, an FXR antagonist, is increased in the model, and decreased with cilofexor treatment, suggesting that cilofexor can out-compete tauro β-muricholic acid for FXR binding.

## Discussion

This study demonstrates that cilofexor improves cholestatic liver injury and decreases hepatic fibrosis, a key driver of liver failure in PSC, and to a certain extent hepatic inflammation, in the *Mdr2*^*-/-*^ mouse model of sclerosing cholangitis. Treatment with cilofexor for 10 weeks led to an improvement in liver fibrosis, as shown by decreased hydroxyproline content at all dose levels and PSR staining at a dose of 90 mg/kg. Cilofexor at a dose of 90 mg/kg decreased inflammatory markers in the liver, as demonstrated by reduced mRNA levels of *Ccl2* and *Cxcl1,* and reduced levels of the cytokines CCL3 and IL-18. Serum levels of AST, ALP, and TBIL were also reduced at 90 mg/kg (Fig. 1, Fig. 2, Fig. 3, Fig. 4). All dose levels of compound administration resulted in expected target engagement with induction of *Shp* and *Fgf15* in the ileum. At 90 mg/kg, the reduced expression of *Cyp7a1*, a key enzyme involved in BA synthesis, resulted in a significant decrease in serum and intrahepatic BA levels. Reduced serum BA levels in mice treated with 10 mg/kg or 30 mg/kg of cilofexor, despite unchanged Cyp7a1, may be explained by the fact that *Cyp7a1* mRNA expression/activity follows the circadian rhythm, peaking at 2 h in the dark phase,^21^ a time point where differences in expression levels may be most distinct.

Our observations that cilofexor decreased liver fibrosis and reduced ALT, AST and TBIL in animals are in line with the findings of a 12-week, randomized, placebo-controlled phase II study in patients with PSC which clearly demonstrated that cilofexor was well tolerated and led to significant improvements in liver biochemistry and serum markers of cholestasis and liver fibrosis.^15^ In a recent 96-week open-label extension of this phase II study of PSC, cilofexor was safe and improved liver biochemistry and biomarkers of cholestasis and cellular injury.^11^ Moreover, cilofexor was also beneficial in a phase II randomized-controlled trial in non-cirrhotic patients with NASH (non-alcoholic steatohepatitis). Over a time-course of 24 weeks, cilofexor improved hepatic steatosis, liver biochemistry, and serum BAs.^22^

Non-steroidal FXR agonists like cilofexor may differ in comparison to the clinically approved OCA in their pharmacokinetic properties and therapeutic mechanisms since their metabolism and transport in the enterohepatic circulation are distinct from endogenous BAs and BA-derived FXR ligands.^23^ Selective pharmacological activation of intestinal FXR has been shown to sufficiently elicit several beneficial metabolic and anti-cholestatic effects.[24], [25], [26] As such, intestinal agonism of FXR and the subsequent increase of gut-derived Fgf15, as well as administration of FGF19 and its mimetics,^24^^,^^27^ all known to suppress BA synthesis, reduced cholestasis in *Mdr2*^*-/-*^ mice.^24^ Furthermore, it has been demonstrated that intra-duodenal but not intravenous BA administration results in reduced hepatic BA synthesis,^28^ indicating that intestinal BA signaling is key in regulating BA synthesis in the liver. These observations are strengthened by our findings, as treatment with cilofexor, the first non-steroidal FXR agonist evaluated for multiple weeks of dosing in in this model, resulted in a profound increase in intestinal *Fgf15* and *Shp* mRNA expression followed by reduced intrahepatic BA levels.

In addition to BA metabolism, hepatic FXR may counteract hepatic inflammation by suppressing NF-κB signaling^29^ and controlling the macrophage-T_H_1/17 axis in the liver.^30^ Using intestinally biased FXR agonists may be a way to dissect the role of intestinal FXR-related repression of BA synthesis from hepatic FXR-related anti-inflammatory effects in the development of cholestatic liver disease in the *Mdr2*^*-/-*^ mouse. Recently, comparison of a systemic *vs*. an intestinally biased FXR agonist revealed that hepatic FXR controls proinflammatory cytokine production via liver-infiltrating immune cells. The authors showed that only treatment with the systemic FXR agonist inhibited innate cytokine production by hepatic macrophages and blocked IL-1β- and TNFα-dependent licensing of T lymphocytes and thus protected from disease progression.^30^ Therefore, the mild anti-inflammatory effect seen in cilofexor-treated *Mdr2*^*-/-*^ mice might be explained by its rather gut preferential effects, yet increased *Shp* mRNA expression in livers of *Mdr2*^*-/-*^ mice at 90 mg/kg cilofexor may suggest some hepatic FXR targeting at this dose level. Last, IL-18 has been identified to correlate positively with the severity of PBC in patients,^31^ was tendentially increased in patients with PSC^32^ and has been shown to increase the risk of liver injury in a non-alcoholic fatty liver disease mouse model.^33^ Although IL-18 may have a negligible role in the *Mdr2*^*-/-*^ mouse, its decreased levels in the liver following cilofexor treatment may be of particular relevance in the human situation.

Of note, mild hepatic FXR agonism of cilofexor could also explain the significantly increased biliary bicarbonate output which was seen in *Mdr2*^*-/-*^ mice treated with the dual FXR/TGR5 agonist INT767 (6-fold more potent to FXR than OCA).^34^ This phenomenon may also, at least in part, contribute to the improvement of the hepatic phenotype seen in *Mdr2*^*-/-*^ mice treated with cilofexor.

Mechanistically, it has been shown that cilofexor treatment in an animal model of NASH reduced activation of HSCs.^35^ This observation is in line with our finding that cilofexor significantly reduced mRNA expression of markers of activated stellate cells, *αSma, Desmin*, and *Pdgfrβ*. Our finding is in accordance with previous studies showing that FXR agonists prevent HSC activation^36^ and that tropifexor (non-steroidal FXR agonist) reduces collagen deposition in HSC/hepatocyte co-cultures.^37^ Furthermore, a direct comparison of OCA with the FXR agonist EDP-305 in *Mdr2*^*-/-*^ mice showed that EDP-305 more potently improved hepatic fibrosis and downregulated activation of HSCs.^38^ Moreover, while activation of FXR in HSCs has been shown to have direct anti-fibrotic effects, increased levels of FGF19 had no pro-fibrotic effect on HSCs.^39^

In summary, treatment of the *Mdr2*^*-/-*^ mouse model of sclerosing cholangitis with the non-steroidal FXR agonist cilofexor improved histological features of sclerosing cholangitis, cholestasis and hepatic fibrosis, indicating that pharmacological stimulation of intestinal FXR-mediated gut-liver signaling via FGF15 (thereby reducing BA synthesis) may be sufficient to attenuate cholestatic liver injury in this mouse model.

## Financial support

This work was supported by a research project grant by Gilead.

## Authors’ contributions

*Study concept and design:* Claudia D. Fuchs, Natalie Sroda, David Hollenback, John Liles, Wesley Minto, Ruchi Gupta, Grant Budas and Michael Trauner. *Manuscript draft:* Claudia D. Fuchs and Natalie Sroda. *Data collection:* Claudia D. Fuchs, Natalie Sroda, Wesely Minto, David Hollenback, Hubert Scharnagl. *Statistical analysis:* Claudia D. Fuchs and Natalie Sroda. *Data interpretation:* Claudia D. Fuchs, Natalie Sroda, David Hollenback, Grant Budas and Michael Trauner. *Critical revision of the manuscript for important intellectual content:* Tatjana Stojakovic, Hubert Scharnagl, David Hollenback, Grant Budas and Michael Trauner. *Manuscript outline and revisions, study oversight, and funding acquisition:* Michael Trauner.

## Data availability statement

Datasets generated during the current study are available from the corresponding authors upon request.

## Conflict of interest

Michael Trauner served as a consultant for Abbvie, Albireo, BiomX, Boehringer Ingelheim, Falk, Gilead, Genfit, Hightide, Intercept, Jannsen, MSD, Novartis, Phenex, Pliant, Regulus, Siemens, Shire and is a member of the speakers’ bureau of BMS, Falk, Gilead, Intercept Madrigal MSD and Roche. He further received travel grants from Abbvie, Falk, Gilead, Intercept, Jannsen and Roche and unrestricted research grants from Alnlyam, Albireo, Cymabay, Falk, Gilead, Intercept, MSD, Takeda and Ultragenyx. He is also co-inventor of a patent on the medical use of norUDCA. Gilead employees own stock in Gilead. Claudia D. Fuchs received travel grants from Gilead, Roche, Falk, Merck, Vifor, Abbvie, and Böhringer Ingelheim. All other authors have no financial disclosures concerning this study to report.

Please refer to the accompanying ICMJE disclosure forms for further details.
