# Supplementary material for: Non-steroidal FXR agonist cilofexor improves cholestatic liver injury in the Mdr2-/- mouse model of sclerosing cholangitis
Source: JHEP Rep. 2023 Aug 3;5(11):100874. doi: 10.1016/j.jhepr.2023.100874 (PMC10568427; doi:10.1016/j.jhepr.2023.100874)
Supplement: Multimedia component 1 [file mmc1.pdf]

Non-steroidal FXR agonist cilofexor improves cholestatic liver injury in the *Mdr2*$^{-/-}$ mouse model of sclerosing cholangitis

Claudia D Fuchs, Natalie Sroda, Hubert Scharnagl, Ruchi Gupta, Wesley Minto, Tatjana Stojakovic, John T. Liles, Grant Budas, David Hollenback, Michael Trauner

Table of contents

Fig. S1……………………………………………………………………..........................3

Fig. S2……………………………………………………………………..........................4

Fig. S3……………………………………………………………………..........................5

Fig. S4……………………………………………………………………..........................6

Fig. S5……………………………………………………………………..........................7

Fig. S6……………………………………………………………………........................8

Table S1 ……………………………………………………………………......................9

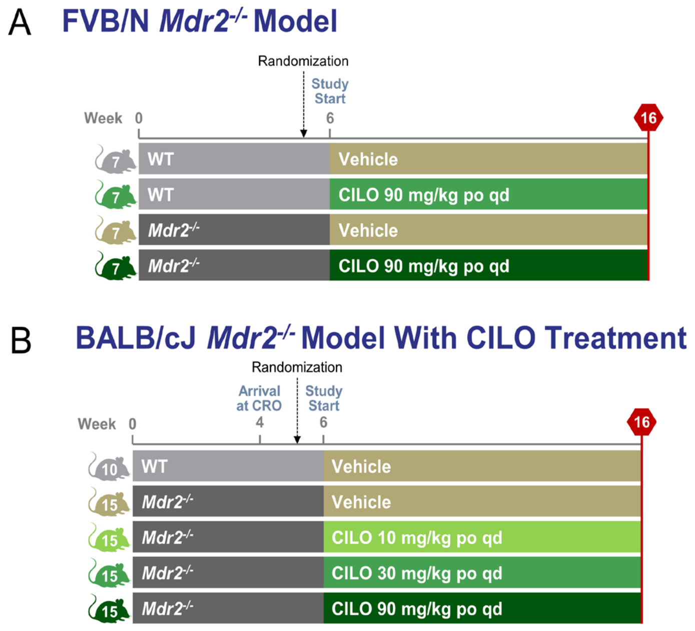

Supporting Figure 1 Fuchs and Sroda et al

Fig. S1: FVB/N and BALB/cJ *Mdr2$^{-/-}$* study designs. (A) Six-week-old male FVB/N *Mdr2$^{-/-}$* mice or WT littermates were treated orally at 0 or 90 mg/kg of cilofexor once per day for ten weeks. (B) Six-week-old male and female BALB/cJ *Mdr2$^{-/-}$* mice were treated orally at 0, 10, 30, or 90 mg/kg of cilofexor once per day for ten weeks. BALB/cJ WT littermates were treated orally once per day with vehicle and used as the control. Abbreviations: WT, wild type; *Mdr2$^{-/-}$*, multi-drug resistance protein 2 knock out; CILO, cilofexor.

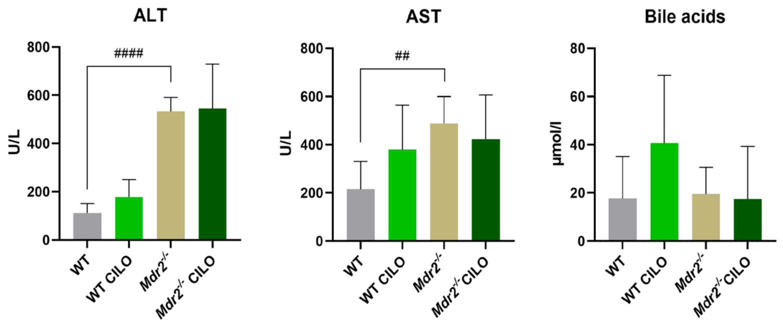

Supporting Figure 2, Fuchs and Sroda et al

Fig. S2: Serum clinical chemistry parameters are not affected by cilofexor treatment in the FVB/N *Mdr2*$^{-/-}$ model. Serum clinical chemistry markers ALT, AST, and total BAs were unchanged after treatment with cilofexor (90 mg/kg) for ten weeks. Results are expressed as mean ± SD. Abbreviations: WT, wild type; *Mdr2*$^{-/-}$, multi-drug resistance protein 2 knock out; ALT, Alanine amino transferase; AST, Aspartate amino transferase.

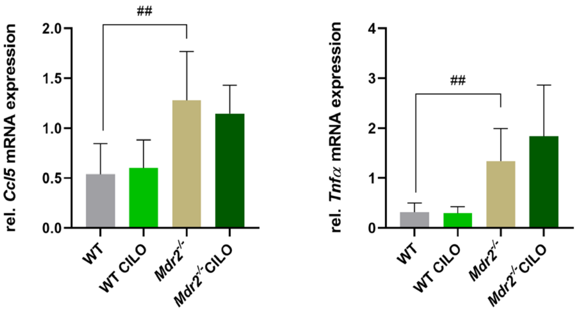

Supporting Figure 3, Fuchs and Sroda et

Fig. S3: Cilofexor has no effect on hepatic inflammatory markers in the FVB/N *Mdr2*$^{-/-}$ model. Expression of hepatic inflammatory genes *Ccl5* and *Tnfα* were unchanged after treatment with cilofexor (90 mg/kg) for ten weeks. Results are expressed as mean ± SD. Abbreviations: WT, wild type; *Mdr2*$^{-/-}$*,* multi-drug resistance protein 2 knock out; *Ccl5*, C-C motif ligand 5; *Tnfα*, Tumor necrosis factor alpha.

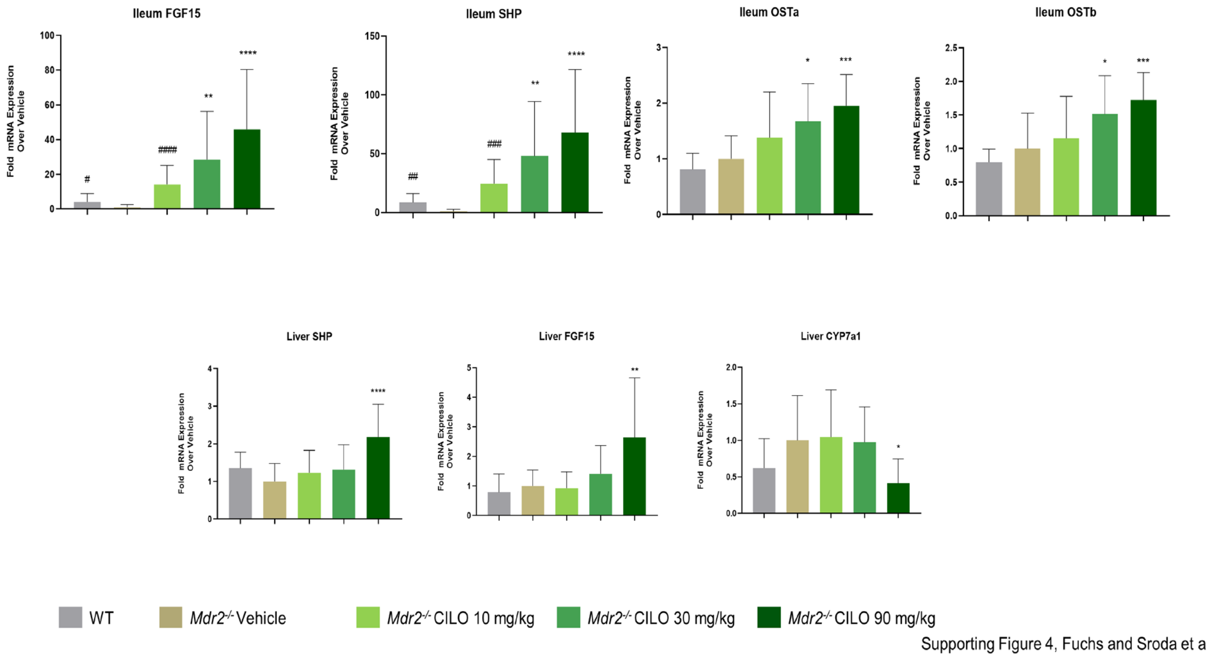

Fig. S4: Ileum and hepatic expression of FXR pathway genes after agonism with cilofexor in the BALB/cJ *Mdr2$^{-/-}$* mouse model. Cilofexor increased iliac expression of *Shp, Fgf15, Ostα,* and *Ostβ* in a dose-dependent manner. Only the highest dose of Cilofexor (90 mg/kg) activated hepatic expression of *Shp* and *Fgf15* and suppression of *Cyp7a1.* Results are expressed as mean ± SD as per figure 1 or 2. Abbreviations: WT, wild type; *Mdr2$^{-/-}$*, multi-drug resistance protein 2 knock out; CILO, cilofexor; *Shp*, small heterodimer partner; *Fgf15*, fibroblast growth factor 15; *Ostα*, organic solute transporter alpha; *Ostβ*, organic solute transporter beta; *Cyp7a1*, Cytochrome P450 family 7 subfamily A member 1.

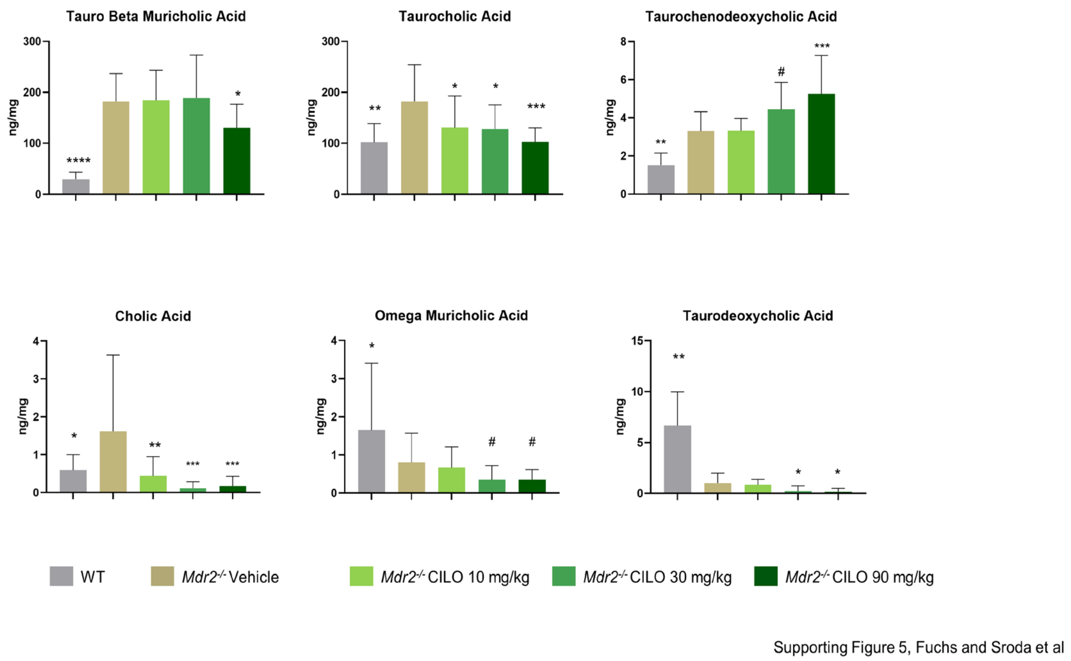

Fig. S5: Effect of cilofexor treatment on selected BA species. Results are expressed as mean ± SD as per figure 2. Abbreviations: WT, wild type; *Mdr2*$^{-/-}$*,* multi-drug resistance protein 2 knock out; CILO, cilofexor.

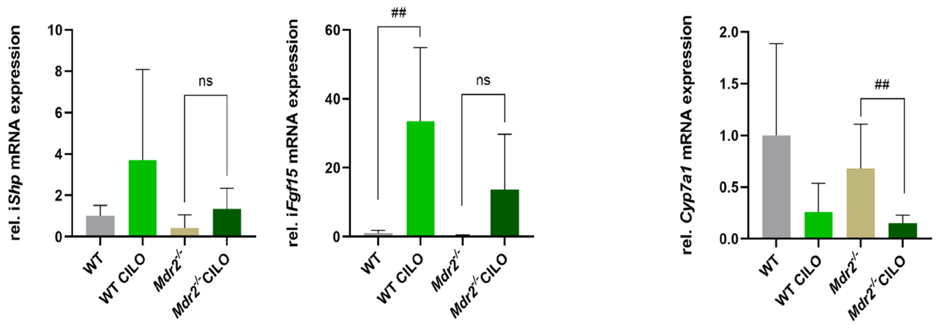

Supporting Figure 6, Fuchs and Sroda et al

Fig. S6: Ileum and hepatic expression of FXR pathway genes after agonism with cilofexor in the FVB/N *Mdr2*$^{-/-}$ mouse model. Cilofexor increased iliac expression of *Shp* and *Fgf15* and suppressed hepatic *Cyp7a1.* Results are expressed as mean ± SD as per figure 1. Abbreviations: WT, wild type; *Mdr2*$^{-/-}$*,* multi-drug resistance protein 2 knock out; *Shp*, small heterodimer partner; *Fgf15*, fibroblast growth factor 15; *Cyp7a1*, Cytochrome P450 family 7 subfamily A member 1.

Table S1: Comparison of FVB/N and BALB/cJ Mdr2$^{-/-}$ mouse models

Parameter	FVB/N	BALB/cJ
PSR	4.47 ± 0.49%	9.09 ± 2.78%
Hydroxyproline	0.39 ± 0.08% total protein	1.20 ± 0.39% total protein
ALP	483 ± 116 U/L	904 ± 209 U/L
AST	488 ± 112 U/L	1269 ± 479 U/L
ALT	533 ± 59 U/L	1178 ± 417 U/L
Serum BAs	20 ± 11 µmol/L	473 ± 300 µmol/L

Abbreviations: *Mdr2$^{-/-}$,* multi-drug resistance protein 2 knock out; ALT, Alanine amino transferase; AST, Aspartate amino transferase; PSR, picrosirius red; BA, Bile acid.
