# Supplementary material for: Non-steroidal FXR agonist cilofexor improves cholestatic liver injury in the Mdr2-/- mouse model of sclerosing cholangitis
Source: JHEP Rep. 2023 Aug 3;5(11):100874. doi: 10.1016/j.jhepr.2023.100874 (PMC10568427; doi:10.1016/j.jhepr.2023.100874)
Supplement: Multimedia component 2 [file mmc2.docx]

**JHEP Reports**

**CTAT methods**

Tables for a “Complete, Transparent, Accurate and Timely account” (CTAT) are now mandatory for all revised submissions. The aim is to enhance the reproducibility of methods.

- Only include the parts relevant to your study
- Refer to the CTAT in the main text as ‘Supplementary CTAT Table’
- Do not add subheadings
- Add as many rows as needed to include all information
- Only include one item per row

**If the CTAT form is not relevant to your study, please outline the reasons why:**

|  |
| --- |

- 1. **Antibodies**

| **Name** | **Citation** | **Supplier** | **Cat no.** | **Clone no.** |
| --- | --- | --- | --- | --- |
| F4/80 |  | Cell Signalling | 70076 | D2S9R |

- 1. **Cell lines**

| **Name** | **Citation** | **Supplier** | **Cat no.** | **Passage no.** | **Authentication test method** |
| --- | --- | --- | --- | --- | --- |
|  |  |  |  |  |  |

- 1. **Organisms**

| **Name** | **Citation** | **Supplier** | **Strain** | **Sex** | **Age** | **Overall n number** |
| --- | --- | --- | --- | --- | --- | --- |
| Mdr2 KO |  | Own colony | FVB/N | male | 8weeks at starting point | 14 |
| WT |  | Own colony | FVB/N | male | 8weeks at starting point | 12 |
| Mdr2 KO |  | JAX | BALB/cJ | Female and male | 6weeks at starting point | 20 |
| WT |  | JAX | BALB/cJ | Female and male | 6weeks at starting point | 75 |

- 1. **Sequence based reagents**

| **Name** | **Sequence** | **Supplier** |
| --- | --- | --- |
| Cyp7a1 fwd | gggattgctgtggtagtgagc | MWG Eurofins |
| Cyp7a1 rev | ggtatggaatcaacccgttgtc | MWG Eurofins |
| Shp fwd | aagggcacgatcctcttcaa | MWG Eurofins |
| Shp rev | gtaccagggctccaagact | MWG Eurofins |
| Tnfa fwd | catcttctcaaaattcgagtgacaa | MWG Eurofins |
| Tnfa rev | tgggagtagacaaggtacaaccc | MWG Eurofins |
| Fgf15 fwd | gaggaccaaaacgaacgaaatt | MWG Eurofins |
| Fgf15 rev | acgtccttgatggcaatcg | MWG Eurofins |
|  |  |  |
| Pdgfrb fwd | actcgcaacatgtctgagac | MWG Eurofins |
| Pdgfrb rev | acattgacctgcagcttgaa | MWG Eurofins |
| Desmin fwd | tcaacttccgagaaaccagc | MWG Eurofins |
| Desmin rev | gtagcctcgctgacaacctc | MWG Eurofins |
| Ccl5 fwd | cctcaccatatggctcggacacca | MWG Eurofins |
| Ccl5 rev | agcgcgagggagaggtaggc | MWG Eurofins |
|  |  |  |
| Ccl2 | Mm00441242_m1 | NanoString Technologies, Inc., Seattle, WA |
| Cxcl1 | NM008176 | NanoString Technologies, Inc., Seattle, WA |
| Cd45 | Mm01293577_m1 | NanoString Technologies, Inc., Seattle, WA |
| Cd68 | NM001291058 | NanoString Technologies, Inc., Seattle, WA |
| Osta | Mm00521530_m1 | NanoString Technologies, Inc., Seattle, WA |
| Ostb | Mm01175040_m1 | NanoString Technologies, Inc., Seattle, WA |
| Fgf15 | Mm00433278_m1 | NanoString Technologies, Inc., Seattle, WA |
| Cyp7A1 | Mm00484150_m1 | NanoString Technologies, Inc., Seattle, WA |
| Shp | Mm00442278_m1 | NanoString Technologies, Inc., Seattle, WA |

- 1. **Biological samples**

| **Description** | **Source** | **Identifier** |
| --- | --- | --- |
|  |  |  |

- 1. **Deposited data**

| **Name of repository** | **Identifier** | **Link** |
| --- | --- | --- |
|  |  |  |

- 1. **Software**

| **Software name** | **Manufacturer** | **Version** |
| --- | --- | --- |
| **Excel** | **windows** | **2013** |
| **Word** | **windows** | **2013** |
| **GraphPad Prism** | **windows** | **8** |

- 1. **Other (*e.g*. drugs, proteins, vectors etc.)**

| **Cilofexor** | **Gilead** |  |
| --- | --- | --- |
|  |  |  |

- 1. **Please provide the details of the corresponding methods author for the manuscript:**

| **michael.trauner@meduniwien.ac.at** |
| --- |

**2.0 Please confirm for randomised controlled trials all versions of the clinical protocol are included in the submission. These will be published online as supplementary information.**

|  |
| --- |
